# McMunn’s Elixir

**Published:** 1864-05

**Authors:** 


### McMUNN'S ELIXIR.

Since the publication in our last Journal of the recipe for making this preparation from the late Dr. James R. Chilton, the celebrated Chemist of

New York, Mr. Wm. H. Peabody, No. 251 and 432 Main street, Buffalo, has manufactured with great care the article, which he supplies at wholesale, or puts up in prescriptions. Having examined specimens, and observed its practical effects, we have no hesitation in saying that we believe it unsurpassed by any preparation of opium, while it is supplied at one-half or one-quarter the expense of the proprietary article. We most heartily recommend it to those who believe that McMunn's Elixir has superiority over other preparations of opium.

